# Sigmoid Volvulus: Diagnostic Modalities and Sigmoid Gangrene

**DOI:** 10.5152/eurasianjmed.2021.21101

**Published:** 2021-06

**Authors:** Sabri Selcuk Atamanalp, Esra Disci

**Affiliations:** Department of General Surgery, Atatürk University School of Medicine Erzurum, Turkey

Dear Editor:

Sigmoid volvulus (SV) is a rare clinical condition in which the sigmoid colon wraps around itself.^1^ Sigmoid gangrene (SG) is a serious complication of SV and it worsens the prognosis.^2^ We report herein our experiment on the diagnosis of SV as well as determination of the SG by evaluating 1,036 cases of SV treated between June 1966 and January 2021, the SV largest series in literature.^3^

In SV, abdominal pain/tenderness, obstipation, and asymmetrical abdominal distention (Figure 1a), which are described as the volvulus triad, are observed in 52%–99% of patients.^1^,^3^ In our evaluation, these clinical features were found in 98.9%, 96.6%, and 92.4% of patients, respectively. Other clinical features are vomiting, hyperkinetic or hypokinetic bowel sounds, empty rectum or melanotic stool, and shock.^1^,^3^ In endemic regions, the determination of the abovementioned features in a middle aged or elderly man is generally suggestive of SV.^3^ Plain abdominal X-ray radiography demonstrating an omega-shaped sigmoid colon with small intestinal air-fluid levels is diagnostic in 25%–90% of patients (Figure 1b)^1^,^3^; this was observed in 68.2% of our patients. Nevertheless, the diagnostic values of computerized tomography (CT) and magnetic resonance imaging (MRI) are generally reported to be over 90%. In CT and MRI, the pathognomonic finding of SV is mesenteric whirl sign arising from rotated sigmoid mesentery in addition to the dilated sigmoid colon and small intestinal air-fluid levels (Figures 1c, 1d).^3^ In our evaluation, the diagnostic accuracy of CT and MRI were 97.3% and 95.6%, respectively. Endoscopic sign of SV is a spiral torsion of the lumen, usually 20–30 cm from the anal verge (Figure 1e). Endoscopy is diagnostic in 76%–100% of patients^3^; this was observed in 98.7% of our patients. When CT, MRI, or endoscopy are not used, SV is easily misdiagnosed as an intestinal obstruction, which generally requires an emergency laparotomy.^1^,^3^

SG, which is an undesirable complication of SV, is observed in 6.1%–93.4% of patients.^1^,^2^,^4^ SG was observed in 286 (27.6%) of our patients. The pathognomonic indicator of SG is melanotic stool, whereas the other clinical and laboratory findings include fever, abdominal guarding/rebound tenderness, hypotension, shock, somnolence, leukocytosis, and metabolic acidosis.^2^,^3^ Endoscopy is the only way to diagnose SG. In endoscopy, SG shows as brown-black mucosa and effluent (Figure 1e). Nevertheless, when SG is strongly suspected or determined before endoscopy, to prevent a bowel perforation or absorption of toxic material, emergency surgery is preferred without performing an endoscopy. In addition, if endoscopy indicates SG, the procedure is terminated and the patient is treated by emergency surgery. In surgery, a brown-black sigmoid colon indicates SG (Figure 1f).^1^–^3^ SG was diagnosed by digital rectal examination, endoscopy, and surgery in 96, 45, and 145 of our patients, respectively. In SV, SG doubles the mortality rate.^2^,^4^,^5^ In our evaluation, the overall mortality rate was 8.3% (86 patients), whereas 23.4% of the patients (67 patients) with bowel gangrene were lost.

As a result, although the accurate diagnosis of SV is important, the determination of SG, a catastrophic complication of SV, is as important as its diagnosis.

## Figures and Tables

**Figure 1. a–f:**
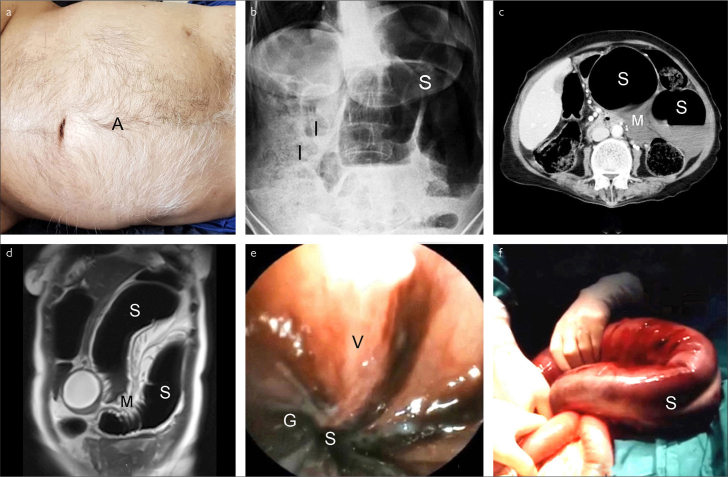
(a) Clinical appearance (A, asymmetrically distended abdomen), (b) Plain erect abdominal X-ray image (S, twisted sigmoid colon; I, intestinal air-fluid levels), (c) Axial abdominal CT image (S, dilated sigmoid colon; M, mesenteric whirl sign), (d) Coronal abdominal MRI image (S, dilated sigmoid colon; M, mesenteric whirl sign), (e) Endoscopic appearance (S, spiral sphincter-like twisted sigmoid lumen; V, viable mucosa; G, gangrenous mucosa), (f) Operative appearance (S, twisted, distended and gangrenous sigmoid colon).
